# Molecular-based approaches to characterize coastal microbial community and their potential relation to the trophic state of Red Sea

**DOI:** 10.1038/srep09001

**Published:** 2015-03-11

**Authors:** Mohd Ikram Ansari, Moustapha Harb, Burton Jones, Pei-Ying Hong

**Affiliations:** 1 Water Desalination and Reuse Center, Environmental Science and Engineering; 2 Red Sea Research Center, King Abdullah University of Science and Technology (KAUST), 4700 King Abdullah Boulevard, Thuwal 23955-6900, Saudi Arabia

## Abstract

Molecular-based approaches were used to characterize the coastal microbiota and to elucidate the trophic state of Red Sea. Nutrient content and enterococci numbers were monitored, and used to correlate with the abundance of microbial markers. Microbial source tracking revealed the presence of >1 human-associated *Bacteroides* spp. at some of the near-shore sampling sites and at a heavily frequented beach. Water samples collected from the beaches had occasional exceedances in enterococci numbers, higher total organic carbon (TOC, 1.48–2.18 mg/L) and nitrogen (TN, 0.15–0.27 mg/L) than that detected in the near-shore waters. Enterococci abundances obtained from next-generation sequencing did not correlate well with the cultured enterococci numbers. The abundance of certain genera, for example *Arcobacter*, *Pseudomonas* and unclassified *Campylobacterales*, was observed to exhibit slight correlation with TOC and TN. Low abundance of functional genes accounting for up to 41 copies/L of each *Pseudomonas aeruginosa* and *Campylobacter coli* were detected. *Arcobacter butzleri* was also detected in abundance ranging from 111 to 238 copies/L. Operational taxonomic units (OTUs) associated with cyanobacteria, *Prochlorococcus,* *Ostreococcus* spp. and *Gramella* were more prevalent in waters that were likely impacted by urban runoffs and recreational activities. These OTUs could potentially serve as quantifiable markers indicative of the water quality.

The shoreline along western Saudi Arabia occupies almost 80% of the eastern Red Sea. Due to the close proximity, cities (e.g. Jeddah, Mecca and Medina) in western Saudi Arabia rely heavily on the Red Sea as the main source for desalinated water. These waters are critical to meet the water demand from the rapidly growing population, as well as for the development of key economic sectors such as marine aquaculture. However, urban development in many of these cities, particularly in Jeddah, has imposed anthropogenic pressures on the coastal water quality. As the population in Jeddah has grown from 2 million in 1993 to 3.25 million in 2010^1^, there has also been a corresponding increase in the volume of municipal wastewater generated. Similar to many other developing countries, infrastructure development has not kept pace with the rapid increase in population. This has resulted in municipal wastewater treatment plants being operated beyond their designed capacities, in turn resulting in discharge of insufficiently treated secondary sewage into the Red Sea.

At the same time, recreational water use at both public and private beaches has increased along the shoreline to the north of Jeddah. These beaches are generally situated in locations where water quality is perceived to be pristine. However, human activities along these beaches provide nonpoint sources of contamination that can impact water quality^2^,^3^. A number of studies have reported the isolation of fecal indicators and human pathogens from marine beach sands, and have observed that the abundance of these microbial contaminants were often detected in higher abundance in sands than in the waters^3^. A portion of these microbial contaminants are readily mobilized and transported in the coastal waters^2^,^4^, adversely affecting water quality. Taken together, anthropogenic sources of contamination arising from sewage discharge and/or recreational activities can result in an excess loading of nutrients and microorganisms to the Red Sea.

Good water quality in the Red Sea is imperative for the continued sustenance of desalination plants. To illustrate, recent studies have noted the importance of source water quality in governing the prevailing trend of membrane fouling and the cost associated with desalination processes^5^,^6^. Good water quality is also essential to ensure minimal occurrence of opportunistic pathogens that can result in public health hazards and economic loss to aquaculture farms in Saudi Arabia. Marine recreational water quality is often monitored based on conventional fecal indicator bacteria like the abundance of enterococci to indicate possible water quality breaches^7^,^8^. However, such culture-based methods are prone to false-negative results that arise from the failure to resuscitate viable but non-culturable cells^9^,^10^. Furthermore, monitoring for fecal indicator bacteria does not provide insights on the possible occurrence of other microbial contaminants (e.g. pathogens).

The advent of molecular-based approaches has provided the ability to address these limitations. To illustrate, quantitative PCR can be used for microbial source tracking by detecting alternative fecal indicators like human-associated *Bacteroides* spp. and animal-specific *Bacteroidales*. Next-generation sequencing enables high-resolution detection of microbial populations that may be present in small abundances, including bacterial genera associated with opportunistic pathogenic species. A positive detection of these genera can be further complemented by quantitative measurements of functional genes associated with pathogens to allow a direct assessment of the potential associated public health hazards. Recent studies of the Red Sea have utilized next-generation sequencing to emphasize the biogeographical patterns of microbial communities in the deep brine systems^11^, as well as in the oligotrophic sites off the Northeastern Red Sea coast or in the central Red Sea^12^,^13^. Although these studies provide unique insights into the endemic microbial communities that are present in the Red Sea, the examined sites are pristine and remain unperturbed by urbanization and anthropogenic contamination.

Molecular-based approaches remain under-utilized in monitoring the coastal Red Sea waters for occurrence of fecal indicators, opportunistic pathogens and other potential microbial markers that may be favored or selected by anthropogenic contamination of marine waters. As such, this study utilizes molecular-based approaches to characterize the coastal microbial community, with emphasis given to detect microbial contaminants and in turn evaluate for the trophic state of these waters. To achieve this aim, samples were collected from sites that were either in close proximities to the sewage treatment plant outfall (i.e., S, NS) or to an urbanized coastal area that receives coastal runoff (i.e., N, T, NS) or to several of the recreational beach facilities (i.e., TW and KW) along the coast (Figure 1).

## Results

### Chemical and microbial quality

The regulatory bodies in Saudi Arabia mandate that total organic carbon (TOC) and total nitrogen (TN) are at less than 15 mg/L and 5 mg/L, respectively, in the marine waters that are adjacent to terrestrial zones^14^. TOC and TN concentration in all water samples fell within the limits of local standards although water sampled from the swash zones of KAUST (KW) and Thuwal (TW) beaches had a higher nutrient content than that in the near-shore waters. To illustrate, the average TOC concentration in KW and TW samples was 1.71 mg/L, while average TN concentration was 0.19 mg/L (Figure 2). The average TOC and TN concentrations in the near-shore waters (i.e., N, S, NS, and T) were 1.13 mg/L and 0.09 mg/L, respectively. The detected TOC and TN concentrations in beach waters were significantly higher than that in all samples collected from the near-shore waters (One-way ANOVA, p < 0.001). Among the near-shore samples, TN concentration in NS samples was significantly higher than that detected in the N sites (One-way ANOVA, p = 0.014) and T sites (One-way ANOVA, p = 0.022) but was not significantly higher than that in the S sites (One-way ANOVA, p = 0.053). For microbial quality, the concentration of enterococci in the local primary contact and secondary contact waters of Saudi Arabia is regulated at less than 40 CFU/100 mL and 200 CFU/100 mL, respectively^14^. In total, seven samples collected from KW and TW exceeded 40 CFU/100 mL (Table 1). In contrast, all near-shore waters, except one collected from S5 at the 5 m depth, had lower than 40 CFU/100 mL of enterococci (Table 1).

### Microbial source tracking

Contamination by human fecal sources was defined as positive when at least two out of the three human-associated *Bacteroides* spp. were present in a single sample (Table 1). Both NS2-5m (i.e., 5 m deep water sample collected at NS2 location) and a TW water sample were positive for all three human-associated *Bacteroides* spp. The detected abundances of *B. vulgatus*, which accounted for up to 1.70 × 10^7^ copies/L, was higher than that detected for *B. uniformis* and *B. fragilis* (Table 2). Similarly, water samples collected at the upper 5 m depth of N1 till N6 locations were positive for two human-associated *Bacteroides*, namely *B. vulgatus* and *B. fragilis*. Beach waters collected from the same location along the Thuwal beach (i.e., TW3) on both sampling occasions were detected positive for *B. vulgatus* (average 4.62 × 10^5^ copies/L), and positive for either *B. uniformis* (4.06 × 10^5^ copies/L) or *B. fragilis* (1.69 × 10^3^ copies/L). The majority of the samples collected at T sites were positive for only 1 human-associated *Bacteroides* spp., while most of the samples collected at S and at KW sites were negative for human-associated *Bacteroides* (Table 1). All samples were tested negative for cow-specific uncultivated *Bacteroidales*.

### Total cell counts and abundance of genera associated with opportunistic pathogens

Flow cytometry cell counts revealed that the water samples collected from N sites and along the beach have an average 7.79 × 10^5^ and 6.44 × 10^5^ cells per mL, respectively. In comparison, the average cells count in S, NS and T sites were 1.04 × 10^6^ cells per mL, which was 45.1% higher than that from N and beach sites (Figure 3A). To approximate the cell abundance of selected genera associated with opportunistic pathogens, we multiplied the relative abundance of individual genera obtained from next-generation sequencing to the total microbial cell counts obtained from flow cytometry. Table 3 showed that the cell abundance of bacterial groups associated with *Acinetobacter*, *Arcobacter*, *Pseudomonas* and unclassified *Campylobacterales* in both KW and TW beach water samples ranged from 2.61 × 10^3^ to 2.47 × 10^6^ cells/L, and these abundances were higher compared to all other near-shore waters (Table 3). In particular, the abundance of *Arcobacter* detected by next-generation sequencing was significantly higher in the TW beach waters than all other samples (One-way ANOVA, p < 0.001). The abundance of *Acinetobacter* in KW waters was also significantly higher than those detected in the near-shore waters (One-way ANOVA, p = 0.002). With the exception of *Acinetobacter* spp., the relative abundance of bacterial populations *Arcobacter*, *Pseudomonas* and unclassified *Campylobacterales* exhibited a slight but significant correlation (Spearman's rank correlation, r_S_ = 0.33–0.44) to the TOC and TN concentrations in the water samples (Table 3). However, only *Arcobacter* and unclassified *Campylobacterales* exhibited a significant correlation with the enterococci MPN (Table 3).

### Quantitative determination of opportunistic pathogenic species

Since *Arcobacter*, *Pseudomonas* and unclassified *Campylobacterales* exhibited positive correlation to the nutrient content in the water samples, qPCR was used to determine if pathogenic species within these genera would be present. Low abundances of functional genes associated with *P. aeruginosa*, ranging from 14 to 41 copies/L, were ubiquitously detected in almost all of the water samples except in the KW beach waters (Figure 3B). The abundance of *C. coli* glyA genes (i.e., 2 to 40 copies/L) was similar to that of *P. aeruginosa* regA gene, but was less frequently detected in the water samples than *P. aeruginosa*. In comparison, *A. butzleri* was detected in an abundance ranging from 111 to 238 copies/L, and was mainly restricted to the water samples collected at TW beach and at the lower depths (i.e., >5 m) of the Red Sea (Figure 3B). Among all samples, the abundances of all tested bacterial species were consistently highest in the TW samples compared to all the other samples.

### Comparative analyses of microbial community

Predominant phyla for all water samples were unclassified Bacteria, *Proteobacteria*, *Cyanobacteria/Chloroplast* and *Bacteroidetes* (Supplementary Figure S1). However, a further analysis based on the relative abundance of genera and unclassified bacterial groups revealed that near-shore seawater samples clustered apart from the beach waters (Figure 4), sharing a similarity of 56% to 61.4%. Furthermore, microbial communities collected at upper and lower depths of the same N, S, NS and T sampling sites shared an average 71%, 68.5%, 72.2% and 77.2% similarity, respectively, with each other. This resulted in an apparent clustering of samples based on upper and lower depths on the multidimensional scaling plot (Figure 4). An OTU-based analysis was conducted to determine the OTUs that likely accounted for the differences. Both KW and TW water samples had unique occurrence for certain OTUs associated with uncultured *Gramella*, *Gammaproteobacteria*, *Deltaproteobacteria* and *Bacteroidetes* (Table 4). In addition, more than 75% of S water samples collected at the upper depths were detected positive for OTUs associated with *Ostreococcus* sp., uncultured *Prochlorococcus* and marine cyanobacterium compared to less than 28.6% of the remaining N, NS, T, KW and TW water samples collected at upper depths (Table 4).

### Microbial contaminants in beach sands

Given that the beach waters differed from the remaining water samples, further assessment was performed to determine the source of differences. A plausible source would be from the beach sands. Heterotrophic plate counts were performed to determine the abundance of heterotrophic bacteria in the sands. The average count of heterotrophic bacteria in KAUST and Thuwal beaches was 6.17 × 10^3^ ± 3.75 × 10^3^ and 2.31 × 10^4^ ± 1.73 × 10^4^ CFU/g of sand. Colonies growing on the heterotrophic plates were isolated based on different morphologies. In total, 57 colonies were isolated and identified for their 16S rRNA gene sequences, and were mainly comprised of *Bacillus* spp. (n = 31) and *Enterococcus* spp. (n = 12). Specifically, near-full length 16S rRNA gene sequencing revealed that the isolates classified under genus *Enterococcus* were *E. faecalis* (n = 3), *E. faecium* (n = 8) and *E. casseliflavus* (n = 1). *Staphylococcus epidermis* (n = 2) was also detected among the heterotrophic bacteria. Because these bacterial genera have been implicated as possible opportunistic pathogens and likely to originate from human hosts, the isolates were further characterized for their resistance profiles to 8 types of antibiotics (e.g. ampicillin, kanamycin, erythromycin, tetracycline, ceftazidime, chloramphenicol, meropenem and ciprofloxacin). With the exception of 4 enterococci isolates and both *Staphylococcus* isolates, all other *Enterococcus* isolates displayed multidrug resistance to at least 2 antibiotics (Supplementary Table S3). These enterococci were therefore likely to be deposited onto the sands during human recreational activities along the beaches, in turn resulting in exceedances in enterococci numbers of the beach waters but not in the near-shore waters.

## Discussion

In this study, molecular methods including qPCR and next-generation sequencing were performed to characterize the coastal microbial community. The trophic quality of the marine waters was evaluated through detection of host-associated fecal indicators and opportunistic pathogenic species. Comparative analyses of the next-generation sequencing data evaluate for the abundance of potential microbial markers at the genus and OTU hierarchical levels. The abundances of the potential markers were then correlated against the nutrient content and enterococci MPN numbers in the water samples to determine the feasibility of using these markers in future studies. Through this complementary approach, our findings suggest that water samples collected from sites with heavy beach usage (e.g. TW) as well as those from sites of close proximity to the urban runoffs (e.g. N1 through N6) would face a higher likelihood of having poor water quality.

The use of human-associated *Bacteroides* had been previously demonstrated on freshwater and marine systems^15^,^16^,^17^,^18^. Similar to a previous study which used the same primer assays to target human-associated *Bacteroides* in the karst groundwater samples^16^, the amplification efficiencies achieved in this study were non-optimal. PCR inhibitors may be present in the DNA templates extracted from the environmental samples which hindered the amplification of gene targets. Alternatively, improved qPCR primer assays should be developed for use in microbial source tracking. Despite the non-optimal amplification efficiencies, Thuwal (TW) beach which is frequented by approximately 200–300 persons per day was positively detected for three human-associated *Bacteroides* spp. in its water samples, suggesting presence of human fecal contamination.

In contrast, molecular-based approaches did not consistently detect the presence of enterococci in the water samples. To illustrate, high-throughput sequencing of the16S rRNA gene amplicons did not detect any *Enterococcus* in most of the water samples except in two of the water samples from KAUST beach at a relative abundance of 0.004–0.006% of the total microbial community. The lack of detection of enterococci by molecular method was in contrast to the occasional exceedances in the cultivable enterococci numbers. It is likely that the detection of enterococci by cultivation and not by molecular-based method may be due to the enrichment of enterococci cells during the cultivation and hence an increased detection sensitivity.

Despite the lack of correlation between the enterococci numbers obtained from molecular-based approaches with that from cultivation, the abundances of selected genera like *Arcobacter, Pseudomonas* and unclassified *Campylobacterales* obtained from high-throughput sequencing showed slight but significant correlations to the total organic carbon, TOC, and total nitrogen, TN (Table 3). In addition, comparative analysis of the sequencing data suggests that beach water samples had unique occurrence for certain OTUs associated with uncultured *Gramella*, *Gammaproteobacteria*, *Deltaproteobacteria* and *Bacteroidetes*. Genomic analysis of a marine *Bacteroidetes* representative *Gramella forsetii* revealed the ability of this bacterium to degrade high molecular weight organic matter^19^, and that the increase in the abundance of marine *Bacteroidetes* have been associated with nutrient-rich water^20^,^21^. A similar observation was made for *Gammaproteobacteria* in marine waters in which an increase in their abundance was observed upon induction by phytoplankton bloom^22^. These findings suggest that the elevated occurrence of the OTUs associated with uncultured *Gramella*, *Bacteroidetes* and *Gammaproteobacteria* in the TW samples may be intricately linked to the increase in TOC and TN in these waters. Considering that these genera were relatively abundant and could be detected by high-throughput sequencing, they can be used as an additional evaluation marker to correlate their abundances with trophic water quality parameters.

Determination of the functional gene copies of the opportunistic pathogenic species within the genera *Pseudomonas*, *Arcobacter* and *Campylobacter* further revealed that the abundance of *P. aeruginosa* and *C. coli* in the marine waters were low. The low abundance of these genera detected by molecular methods may be due to prolonged natural inactivation and decay of the bacterial cells and their respective genomic signatures upon sunlight irradiation^23^,^24^,^25^. Alternatively, these genera may be associated with the big particulate fractions of the seawater and inadvertently removed when filtering the water samples through an 8 μm filter prior to the 0.45 μm filter. Regardless, a relatively higher abundance of microaerophilic *A. butzleri* was detected in most of the water samples collected at the lower depths (i.e., >5 m) of Red Sea where no human-associated *Bacteroides* markers were detected. Together with a past study that detected *Arcobacter* spp. as members of the seawater microbiota that are associated with zooplankton in the coastal environment of Italy^26^, the detection of *A. butzleri* in this study suggests that *A. butzleri* are likely to be indigenous members of the seawater microbiota in the Red Sea. However, it remains unknown if these non-host-associated microorganisms would impose any health risks to sea users, particularly when the presence of this emerging pathogen^27^ does not correlate with the presence of conventional and alternative fecal indicators.

Through the use of remote sensing, a recent study modeled surface wind speeds and the resulting intrusion of surface water in the northern Red Sea^28^. During both summer (mid-May till mid-September) and winter (mid-December till mid-March) seasons, surface wind speeds can reach up to 8 m/s in the southwards direction. Coupled with the high wind speed, eddies in winter can transfer nutrients from the coast to the open waters of the central Red Sea, possibly accounting for the sporadic picoplankton blooms during these seasons^28^. As sampling of the near-shore waters was conducted in April for this study, the lack of detection of most of the microbial markers in the near-shore water samples may be due to the weaker eddies after the winter peak^29^. Alternatively, fringing reefs in the Red Sea may have also hindered intermixing of water from the coast to near-shore regions^30^. Future efforts should aim to increase sampling numbers throughout the year to account for seasonal variations, and to expand molecular-based detection to enteric viruses and/or other waterborne pathogens so as to facilitate a more thorough assessment on the quality of marine waters and the associated public health risks.

## Conclusion

In summary, a wide suite of molecular-based approaches were complementarily used to detect for alternative fecal indicators, genera associated with opportunistic pathogens, functional genes of opportunistic pathogenic species and microbial OTUs that correlate in their abundance with the nutrient contents. Using these approaches, potential contamination arising from urban runoffs and recreational activities were identified at specific sites along the eastern coast of the Red Sea. There was no apparent correlation between the enterococci abundance depicted using molecular and culture-based approaches. Nor was there any apparent correlation of enterococci with the abundance of alternative fecal indicators such as human-associated *Bacteroides* and opportunistic pathogenic species. However, other microbial markers that showed significant correlation with water quality parameters can be detected by molecular-based approaches, and could potentially serve as quantifiable markers indicative of the water quality. These microbial markers include *Arcobacter*, *Pseudomonas*, *Campylobacter* and/or uncultivated OTUs associated with *Gramella*, *Gammaproteobacteria*, *Deltaproteobacteria* and *Bacteroidetes*.

## Methods

### Potential anthropogenic contamination sources

**Figure 1:**
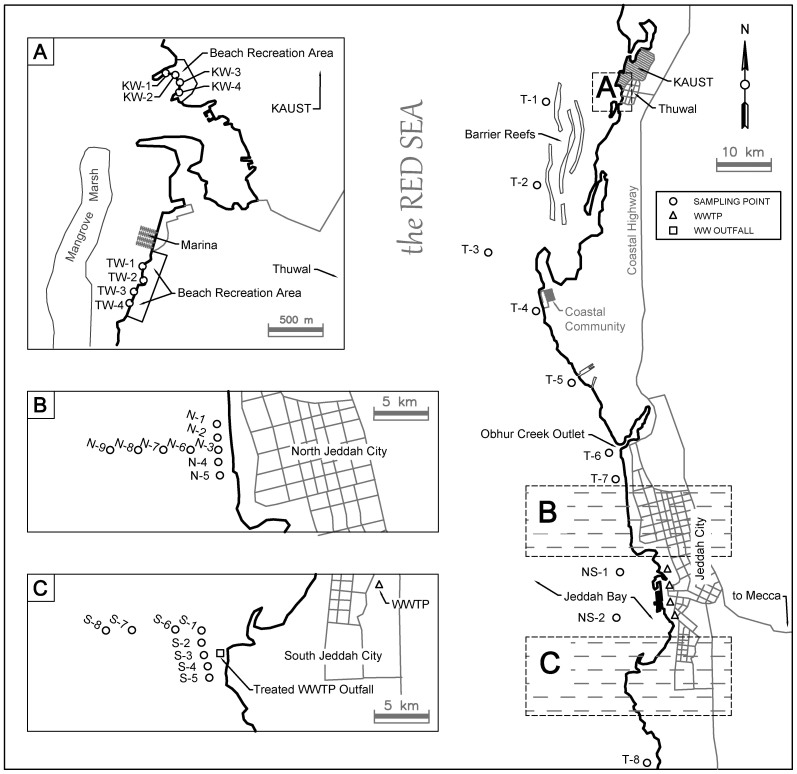
Sampling locations and potential sources of anthropogenic contamination. (A) Sampling points along the KAUST beach (KW1 through KW4) and Thuwal beach (TW1 through TW4), (B) Sampling points N1 through N9 that are at north of Jeddah City, and of close proximity to the urban settlement (C) Sampling points S1 through S8 which are at south of Jeddah City, and of close proximity to a treated wastewater outfall. Sampling points NS1 and NS2 denote sampling sites between north-south sampling sites. Sampling points T1 through T8 denote sampling sites along the coastline. Detailed GPS coordinates are provided as supplementary information. Figure 1 is generated by AutoCAD version 2015 on a commercial license issued to KAUST.

**Figure 2:**
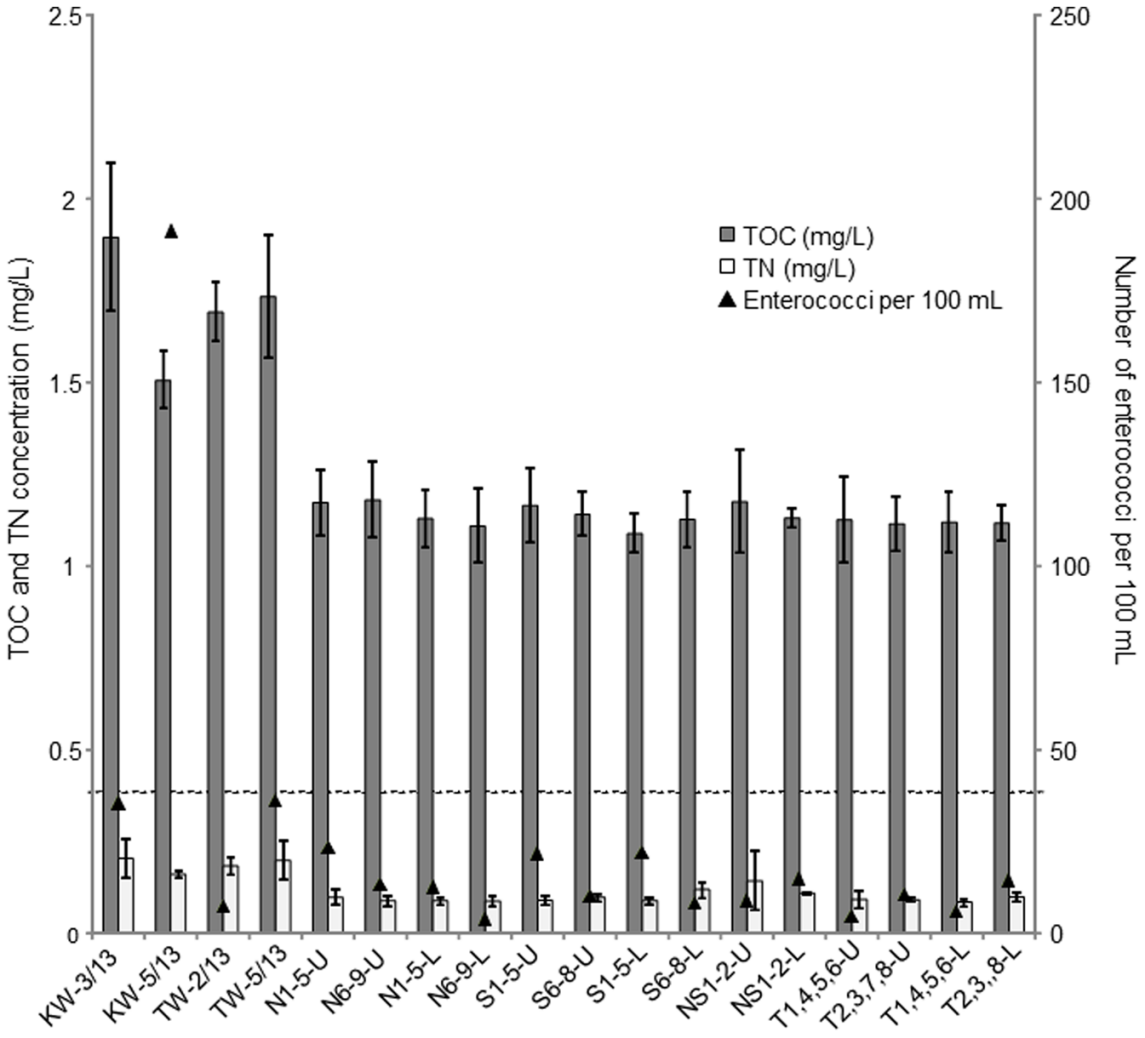
Water quality at the different sampling locations. TOC and TN concentrations of TW and KW were significantly different compared to the other water samples. No significant differences were observed between both upper and lower depths of sampled waters. Bottom dashed line denotes the permissible level of 40 CFU of enterococci per 100 mL of marine water.

**Figure 3:**
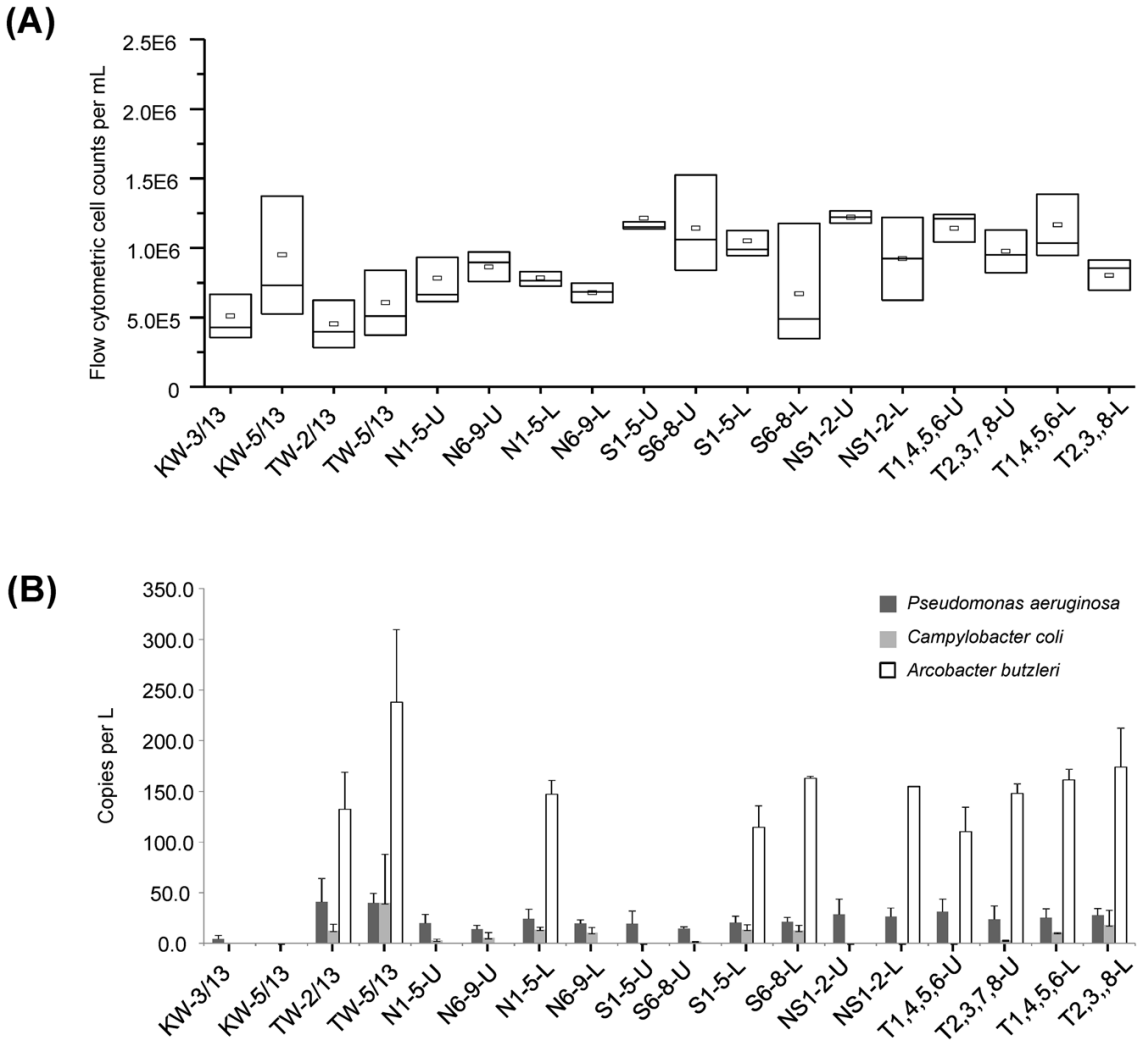
Cell abundances in the water samples as determined by flow cytometry. (A) Total cell counts per mL in the KW and TW beach waters, and in the near-shore waters, and (B) Copies per L of regA, glyA and rpoB associated with *Pseudomonas aeruginosa*, *Campylobacter coli* and *Arcobacter butzleri*, respectively.

**Figure 4:**
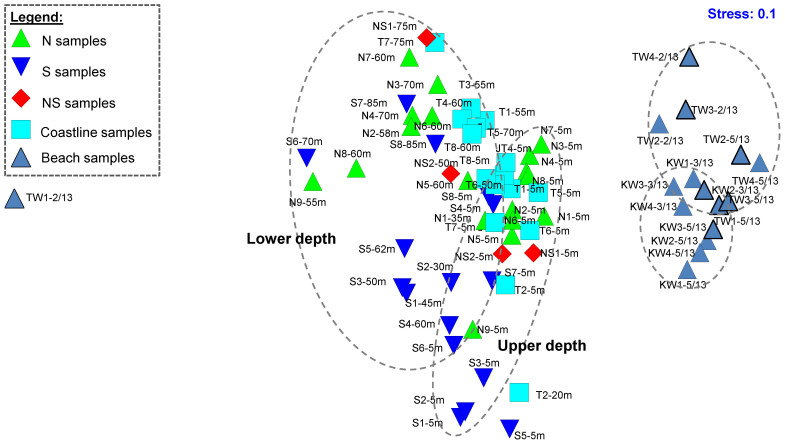
Multidimensional scaling (MDS) plot for the microbial community in N, S, NS, T and beach (KW and TW) waters. Relative abundance of genera and unclassified bacterial groups revealed that near-shore seawater samples clustered apart from the beach waters, and between the upper and lower depths of near-shore sites.

Jeddah is located on the west coast of Saudi Arabia, and is the second most populous city with an estimated 3.25 million residents. The city has a total of approximately 12 municipal wastewater treatment plants (WWTP) which discharged an approximate 299,100 m^3^/d of secondary or tertiary treated wastewater into the Red Sea^31^. Six of these municipal treatment plants can be found along the coast. Discharge of treated municipal wastewater is routed through pipelines that connect from the WWTP plants to the Red Sea. One of these pipelines (39.0741° longitude, 22.2963° latitude) is situated about 500 m from the shore and 10 m below the sea surface^32^. In addition, a small-scale WWTP that utilized an aerobic membrane bioreactor to treat sewage generated from a 5000-persons community within the KAUST campus intermittently discharged tertiary treated effluent that is blended with 97.7% v/v brine through a 2.8 km pipeline into the Red Sea (V. Paterino, personal communication). Several beaches, including the Thuwal and KAUST beaches, can be found north of Jeddah. Thuwal beach is a public beach that is frequented by an estimated 200 to 300 persons per day. The KAUST beach is a private beach that serves the community within the university, and is estimated to be frequented by 40 to 50 persons per day. The sampling sites and potential sources of contamination are illustrated on Figure 1 which is generated by AutoCAD version 2015 commercial license.

### Sampling sites and collection methods

To collect the near-shore waters, a KAUST Red Sea Sampling Expedition was conducted from 23 March till 5 April 2013 where the boat cruised along the Red Sea at 1.5 km near -shore (Figure 1). GPS coordinates of sampling sites were detailed in Supplementary Table S1. Near-shore water samples were collected at four regions, denoted as north (N), south (S), north-south (NS) and coastal transect (T). At each sampling location, 7 L of seawater samples were respectively collected from the upper (5 m from sea surface) and lower (20 m to 85 m from sea surface) depths as an earlier study has shown different chlorophyll densities at these two sampling depths^28^. Specifically, the water samples collected at lower levels (i.e., >5 m) tend to have higher chlorophyll content than that collected at 5 m depths, although it is uncertain if the higher chlorophyll content would be related to anthropogenic contamination and algae bloom. The sampling sites were further chosen to represent sites that are parallel to the coast and presumably more impacted by the contamination sources, as well as sites that are perpendicular to the coast and presumably less impacted by the contamination sources. A rosette sampler equipped with Niskin bottles was deployed from the sampling boat to collect the water samples at each depth. The seawater samples were immediately processed on the boat by filtering through 8 μm polycarbonate filters (EMD Millipore, Germany) to remove suspended particulates. The filtrates were then filtered through 0.45 μm polycarbonate filter (EMD Millipore, Germany) to retain the microbial biomass. All filters were stored in −20°C prior total DNA extraction. Coastal beach water samples were also collected from Thuwal beach and KAUST beach (Figure 1). Two sampling trips were conducted to the KAUST beach (March 2013 and May 2013), and two trips to the Thuwal beach (Feb 2013 and May 2013). Four different sampling points were identified along the swash zones of each beach for the collection of water and sand samples. In total, 16 grab water samples, each of 7 L volume (n = 8 from each beach) were collected from both beaches. In total, 16 beach sand samples were collected. For each sand sample, 2 kg of sands composite was prepared by mixing sands from twelve points within a 1 × 1 m grid. All samples collected from the beaches were brought back on ice immediately to the laboratory for further enumeration of bacterial counts, filtration and total DNA extraction.

### Water quality analyses

Concentrations of total organic carbon and total nitrogen were measured by the high-temperature catalytic oxidation (HTCO) method using a commercially available automatic TOC-V_CPH_ analyzer (Shimadzu, Japan). Samples were filtered through 0.45 μm filters and used for analysis. One blank deionized water and positive control of known TOC and TN concentration were analyzed along with the samples. The TOC-V_CPH_ analyzer with a TNM-1 add-on is routinely used in conjunction with a Shimadzu ASI-V autosampler in non-purgable organic carbon (NPOC) mode, following the methodology recommended by Shimadzu. For flow cytometric analysis on Accuri C6 flow cytometer (BD Biosciences, San Jose, CA), 700 μL of water samples were stained with 7 μL of SYBR Green I (1:100 dilution in DMSO) (Invitrogen, Carlsbad, CA), and incubated in the dark for 10 min at 35°C before measurement. Where necessary, samples were diluted accordingly to achieve less than 4000 events/s at a pre-set flow rate of 35 μL/min.

### Enumeration of enterococci

The number of enterococci in all samples was estimated by five tubes Most Probable Number (MPN) method, with protocols as described in standard method 9230B^33^. Briefly, 10 g of the composite sand was first suspended in 90 mL of normal saline solution (i.e., 0.9% w/v NaCl solution), vortexed, and left to stand for 10 min to allow sand particles to settle. 10 mL, 1 mL and 0.1 mL inoculum from the suspension and water samples were then individually inoculated in azide dextrose broth and incubated for 24 h at 35°C. Tubes showing positive growth were confirmed on bile esculin azide agar for enterococci and examined for typical colonies (i.e., brownish-black colonies with brown halos).

### Isolation of heterotrophic bacteria

Aerobic halophilic and non-halophilic heterotrophic bacteria were cultured on marine agar and nutrient agar, respectively. The plates were incubated at 35°C for 2 d, and different fast growing bacterial isolates with distinct colony morphology were isolated and purified by repeated streaking on the appropriate agar plates.

### Antibiotic resistance tests

Bacterial isolates were tested for their resistance profiles to 30 μg/mL each of ampicillin, kanamycin, erythromycin and tetracycline, as well as 8 μg/mL each of ceftazidime, ciprofloxacin, chloramphenicol and meropenem. To assess the extent of resistance or susceptibility of bacterial isolates to antibiotics, isolates were individually grown in the presence of each of the antibiotics and in the absence of any antibiotics to serve as a control. The optical density of the growth medium at wavelength 600 nm (OD_600_) was subsequently measured using the Spectromax 340pc microplate spectrophotometer (Molecular Devices, Sunnyvale, CA). The threshold value to determine the status of a bacterial isolate as resistant to the antibiotic was set at >50% of the control OD_600_ value.

### Total DNA extraction and sequencing

All water samples which were filtered on 0.45 μm polycarbonate membranes were extracted for their total DNA using the UltraClean® Soil DNA Isolation Kit (MoBio, Carlsbad, USA) with slight modifications. Slight modifications to the manufacturer's protocol were made to ensure a representative extraction of bacteria and archaea populations present in the samples^34^. In brief, 12 μL of 100 mg/mL lysozyme and 1 mg/mL achromopeptidase were added to the extraction buffer, and the sample mixture was incubated at 37°C for 1 h before total DNA extraction. Lysozyme breaks the β-1,4-glycosidic bonds between N-acetylglucosamine and N-acetylmuramic acid in peptidoglycan of most bacteria^35^. Achromopeptidase is added to achieve enzymatic lysis of gram-positive bacteria that are resistant to lysozyme^36^. The addition of lysozyme and achromopeptidase serve to provide enzymatic lysis which will complement the chemical and physical lysis provided by the commercial kit. To perform amplicon next-generation sequencing, the total DNA was amplified for the V4–V5 region of 16S rRNA genes with universal forward 515F: (5′-Barcode-GTGYCAGCMGCCGCGGTA-3′) and reverse 909R: 5′-CCCCGYCAATTCMTTTRAGT-3′) primers. PCR reaction mixtures comprised 10 ng of DNA, 25 μL of Premix F (Epicentre Biotechnologies, WI, USA), 200 nM (each) of barcoded forward and reverse primers, 0.5 U of Ex *Taq* DNA polymerase (Takara Bio, Japan), and the volume added up to 50 μL with molecular-biology grade water. PCR with 30 cycles of thermal program (denaturation, 95°C for 30 s; annealing, 55°C for 45 s; and extension, 72°C for 60 s) was performed. To identify the phylogenetic identities of bacterial isolates, near full-length of 16S rRNA genes were amplified using modified forwarded primer 11F (5′-GTTYGATYCTGGCTCAG-3′) and reverse primer 1492R (5′-GGYTACCTTGTTACGACTT-3′). All amplicons were gel-excised, concentrated and purified with Wizard SV Gel and PCR Clean-up purification kit (Promega, Madison, WI, USA). The concentrations were then measured by Qubit fluorometer (Invitrogen, Carlsbad, CA). Amplicons were submitted to KAUST Genomics Core lab for Ion Torrent PGM sequencing on a 314 chip, and for Sanger sequencing on an Applied Biosystems 3730xl capillary sequencer.

### Next-generation sequencing analyses

Raw sequence reads were first trimmed for their barcode, adaptor and primer sequences. Trimmed sequences were then checked for their quality by removing reads that are <250 nt in length and with Phred score <20. Chimeras were identified on UCHIME^37^ by referencing a core reference set that was downloaded from Greengenes (i.e., gold strains gg16 – aligned.fasta). A total of 1369,708 16S rRNA sequences were obtained after the quality check for all 70 samples collectively. RDP Classifier was used for taxonomical assignments of the 16S rRNA gene sequences at 95% confidence level^38^. Sequences were aligned using the RDP Infernal Aligner, and aligned sequences were binned for unique operational taxonomic units (OTUs) identified at 97% 16S rRNA gene similarity. The cluster matrix generated from RDP pipeline was then used in rarefaction analysis. Rarefaction curves were generated and shown in Supplementary Figure S2 and Figure S3. Microbial richness for each sample was denoted from the rarefaction curves based on a defined sequencing depth of 7,000 sequences. To further perform an OTU-based analysis, all chimera-removed fasta files were combined together with an in-house written Perl Script. The combined sequence file was then identified for the unique OTUs at 97% 16S rRNA gene similarity using CD-Hit (Li and Godzik, 2006). The output file denotes the abundance of each unique OTUs in each barcoded sample, and the nucleotide sequence of each unique OTU. To identify the phylogenetic affiliation of the OTU, the nucleotide sequence was BLASTN against the NCBI database.

### Quantitative PCR

Quantitative PCR (qPCR)-based approach was used for microbial source tracking of host-associated fecal indicators and to quantify the abundance of pathogenic species. Microbial source tracking was performed using three primer pairs that target the human-associated *Bacteroides vulgatus*, *Bacteroides uniformis* and *Bacteroides fragilis*, a primer pair that targets cow-specific uncultivated *Bacteroidales*^18^. Primer assays, targeting toxin A synthesis regulating gene (regA) of *Pseudomonas aeruginosa*^39^, serine hydroxymethyltransferase gene (glyA) of *Campylobacter coli*^40^ and β-subunit of bacterial RNA polymerase rpoB gene of *Arcobacter butzleri*^41^ were also included. Oligonucleotide sequences of the primer assays were listed in Table S2. Gene inserts were obtained from *B. vulgatus* BCRC12903, *B. uniformis* JCM5828, *B. fragilis* BCRC10619, *P. aeruginosa* DSM1117, *C. coli* ATCC 33559 and *A. butzleri* ATCC49616 and from a cow-specific uncultivated *Bacteroidales* clone obtained from an earlier study^18^. qPCR standards were prepared by first cloning the gene inserts into pCR4 TOPO vector (Invitrogen, Carlsbad, CA, USA). Plasmid DNA was extracted using PureYield™ Plasmid Miniprep System (Promega, Madison, WI, USA). The extracted plasmids were sequenced to verify the oligonucleotide sequences of gene inserts, and quantified for their copy numbers per μL. Amplifications to obtain standard curves were performed in triplicate, while test amplifications and negative blanks were run in duplicates. Each reaction volume of 20 μL contained 10 μL of FAST SYBR Green master mix, 0.4 μL of each primer (10 μM), 1 μL of DNA template and 8.2 μL molecular biology grade water. The Applied Biosystems 7900 HT Fast protocol was used for thermal cycling. The protocol includes 40 cycles of 1 s denaturation at 95°C and 60 s of annealing and extension. Dissociation curve analysis was included to detect non-specific amplification. The amplification factors of the standards are listed in Table S2.

### Statistical method

One-way ANOVA was conducted to determine the statistical significance of differences among the means of different sample datasets. t-test was performed using a 2-tailed distribution and unequal variance test. Spearman's rank correlation (r_S_) analysis was used for non-parametric correlation determination. Non-metric multidimensional scaling (nMDS) was performed by Primer-E v5.2.4 based on a Manhattan distance matrix. Data generated from next-generation sequencing was pre-treated by first determining the relative abundance of each individual classified genera and unclassified groups in every sample. This normalization step was required as the sequencing depth differed among the samples (Supplementary Figure S2), and therefore comparison among samples could only be made on the basis of relative abundance of each classified genera and unclassified groups collated from RDP Classifier at 95% confidence level. Subsequently, the normalized data were square-root transformed so as to down-weight the dominant taxa and to allow for the rarer species that were commonly detected in the high-throughput sequencing to exert some influence on the calculation of the similarity matrix^42^.

### Nucleotide sequence accession numbers

All Ion Torrent sequencing files were deposited in the Short Read Archive (SRA) of the European Nucleotide Archive (ENA) under study accession number PRJEB5835. Near-full length 16S rRNA gene Sanger-based sequences were deposited in NCBI database under accession number KJ571203-KJ571216.

## Author Contributions

M.I.A., P.Y.H. and B.J. conceived and designed the experiments; M.I.A., M.H. and P.Y.H. performed the sampling and experiments; M.I.A. and P.Y.H. analyzed the data; P.Y.H. and B.J. contributed reagents/materials/analysis tools; M.I.A., M.H. and P.Y.H. contributed to the writing of the manuscript and preparation of figures.

## Supplementary Material

Supplementary Information Supplementary Information

## Figures and Tables

**Table 1:** Occurrence of human-associated *Bacteroides* spp., *Enterococcus* spp. and cow-specific uncultivated *Bacteroidales* in the water samples. * denotes that water samples tested positive for 2 or more markers were likely to be contaminated by fecal sources originating from human hosts

		Human-associated *Bacteroides* spp.				*Enterococcus*	Cow-specific
Sample sites	Number of samples	Positive for 3 markers*^(sample name)^	Positive for 2 markers*^(sample name)^	Positive for 1 marker	Positive for 0 marker	Exceedances of >40 MPN/100 mL (Culture)	Positive for 0 marker
N	18	0	5^(N1, N3, N4, N5, N6)^	6	7	0	18
S	16	0	0	7	9	1	16
NS	4	1^(NS2)^	0	2	1	0	4
T	16	0	0	12	4	0	16
KW	8	0	0	1	7	5	8
TW	8	1^(TW4-2/13)^	2^(TW3-2/13 and TW3-5/13)^	2	3	2	8

**Table 2:** Abundance of human-associated *Bacteroides* spp. in water samples positive for two and more of the markers. SE denotes standard error. ND denotes not detected

	*B. vulgatus*	*B. uniformis*	*B. fragilis*
		Copies/ng DNA ± SE	
		*Copies/L water* ± SE	
**Positive for 3**			
NS2-5m	1.79 × 10^4^ ± 5.51 × 10^3^	4.55 × 10^2^ ± 2.03 × 10^1^	8.51 ± 3.68 × 10^−1^
	*3.03* × *10^6^ ± 9.32* × *10^5^*	*7.70* × *10^4^ ± 3.43* × *10^3^*	*1.44* × *10^3^ ± 6.23* × *10^1^*
TW4-2/13	6.15 × 10^4^ ± 4.09 × 10^3^	2.88 × 10^4^ ± 2.47 × 10^2^	6.07 × 10^2^ ± 8.82 × 10^1^
	*1.70* × *10^7^ ± 1.13* × *10^6^*	*7.95* × *10^6 ^± 6.81* × *10^4^*	*1.67* × *10^5^ ± 2.43* × *10^4^*
**Positive for 2**			
N1-5m	6.34 × 10^3^ ± 3.76 × 10^2^	ND	5.35 ± 2.44
	*7.83* × *10^5^ ± 4.64* × *10^4^*		*6.61* × *10^2^ ± 3.02* × *10^2^*
N3-5m	5.34 × 10^3^ ± 7.36 × 10^2^	ND	4.79 × 10^1^ ± 3.49 × 10^1^
	*1.04* × *10^6^ ± 1.43* × *10^5^*		*9.33* × *10^3^ ± 6.79* × *10^3^*
N4-5m	2.39 × 10^4^ ± 6.58 × 10^2^	ND	2.66 × 10^1^ ± 2.59
	*2.24* × *10^6^ ± 6.16* × *10^4^*		*2.49* × *10^3^ ± 2.42* × *10^2^*
N5-5m	1.46 × 10^4^ ± 1.47 × 10^3^	ND	1.24 × 10^1^ ± 1.94
	*2.16* × *10^6^ ± 2.17* × *10^5^*		*1.83* × *10^3^ ± 2.85* × *10^2^*
N6-5m	1.55 × 10^4^ ± 2.94 × 10^3^	ND	2.85 ± 10^1^ ± 1.08 × 10^1^
	*2.58* × *10^6^ ± 4.88* × *10^5^*		*4.74* × *10^3^ ± 1.79* × *10^3^*
TW3-2/13	1.59 × 10^3^ ± 2.00 × 10^2^	ND	1.07 × 10^1^ ± 4.66 × 10^−1^
	*2.52* × *10^5^ ± 3.16* × *10^4^*		*1.69* × *10^3^ ± 7.36* × *10^1^*
TW3-5/13	4.06 × 10^3^ ± 2.95 × 10^2^	2.49 × 10^3^ ± 1.32 × 10^2^	ND
	*6.71* × *10^5^ ± 4.88* × *10^4^*	*4.06* × *10^5^ ± 2.16* × *10^4^*	

**Table 3:** Average relative abundance and approximated cell numbers of selected genera associated with opportunistic pathogens. Relative abundances were correlated to the total organic carbon (TOC), total nitrogen (TN) and enterococci MPN numbers

	Average relative percentage abundance in samples collected from sites (Approximated cells per L)						
Selected genera associated with opportunistic pathogens	KW	TW	N1-N6 at 5 m depth	All remaining sites	Correlation to TOC	Correlation to TN	Correlation to enterococci MPN number
*Acinetobacter*	0.564% (2.47 × 10^6^)	0.111% (3.92 × 10^5^)	0.032% (2.48 × 10^5^)	0.032% (2.74 × 10^5^)	r_S_ = 0.127 (p = 0.296)	r_S_ = 0.263 (p = 0.028)	r_S_ = −0.094 (p = 0.444)
*Arcobacter*	0.002% (1.78 × 10^4^)	0.020% (9.34 × 10^4^)	0.003% (1.25 × 10^4^)	0.0003% (3.23 × 10^3^)	r_S_ = 0.391 (p < 0.001)	r_S_ = 0.401 (p < 0.001)	r_S_ = 0.267 (p = 0.028)
*Pseudomonas*	0.007% (4.60 × 10^4^)	0.001% (2.61 × 10^3^)	Not detected	0.0002% (1.91 × 10^3^)	r_S_ = 0.348 (p = 0.003)	r_S_ = 0.326 (p = 0.006)	r_S_ = 0.156 (p = 0.205)
Unclassified *Campylobacterales*	0.001% (9.17 × 10^3^)	0.003% (1.36 × 10^4^)	Not detected	Not detected	r_S_ = 0.438 (p < 0.001)	r_S_ = 0.408 (p < 0.001)	r_S_ = 0.263 (p = 0.030)

**Table 4:** Comparison of abundance of operational taxonomic units (OTUs) detected at different sampling sites. OTUs were identified for their best-matched phylogenetic affiliation. * All OTUs denoted below were significantly different by t-test in the average relative abundance between both compared sets

	Percentage occurrence, % (Average relative abundance, %)*			
OTU name	KW and TW	All other samples	Best match identity and accession number	Similarity, E-value
OTU129	75 (0.011)	Not present	Uncultured bacterium clone (JQ347438)	100%, 2E-122
OTU172	93.75 (0.038)		Uncultured *Bacteroidetes* clone (AM238576)	99%, 0
OTU320	81.25 (0.011)		Uncultured bacterium (JQ062604)	94%, 5E-162
OTU1331	87.5 (0.009)		Uncultured marine bacterium (FJ951115)	99%, 0
OTU2934	75 (0.009)		Uncultured gamma proteobacterium (AM229490)	96%, 3E-177
OTU4131	75 (0.010)		Uncultured bacterium (EU283113)	94%, 8E-159
OTU4377	75 (0.008)		Uncultured bacterium (JQ195446)	93%, 2E-153
OTU5970	75 (0.011)		Uncultured *Gramella* (EU328069)	98%, 0
OTU6877	87.5 (0.020)		Uncultured bacterium (HQ601704)	95%, 1E-168
OTU7311	75 (0.006)		Uncultured *Bacteroidetes* (HQ241995)	92%, 3E-157
OTU7390	75 (0.012)		Uncultured delta proteobacterium (FM211795)	98%, 0
OTU8700	87.5 (0.017)		Uncultured bacterium (EU010232)	98%, 0
OTU14701	75 (0.020)		Uncultured bacterium (HQ601704)	99%, 0
	**S-5m samples**	**N-5m, NS-5m, T-5m, KW and TW water samples**		
OTU1929	75 (0.009)	28.57 (0.003)	Uncultured marine bacterioplankton (KC003326)	97%, 0
OTU2554	75 (0.010)	17.14 (0.001)	*Prochlorococcus* sp. (HQ675339)	94%, 2E-166
OTU4618	75 (0.004)	20.00 (0.001)	Uncultured bacterium clone (EU035867)	94%, 8E-159
OTU4905	75 (0.005)	20.00 (0.001)	*Ostreococcus* sp. RCC393 (AY702161)	97%, 0
OTU6685	75 (0.006)	22.86 (0.002)	Uncultured marine cyanobacterium (HQ540323)	98%, 0
OTU10157	87.5 (0.005)	17.14 (0.001)	Uncultured bacterium clone (EF574538)	97%, 5E-180
OTU12972	87.5 (0.008)	17.14 (0.001)	Uncultured bacterium clone (EF575131)	96%, 8E-178
OTU21585	87.5 (0.006)	28.57 (0.002)	Uncultured bacterium clone (EU800551)	96%, 3E-164
