# Supplementary material for: Molecular-based approaches to characterize coastal microbial community and their potential relation to the trophic state of Red Sea
Source: Sci Rep. 2015 Mar 11;5:9001. doi: 10.1038/srep09001 (PMC4355682; doi:10.1038/srep09001)
Supplement: Supplementary Information [file srep09001-s1.pdf]

Molecular-based approaches to characterize coastal microbial community and their potential relation to the trophic state of Red Sea

Mohd Ikram Ansari [1], Moustapha Harb [1], Burton Jones [2], and Pei-Ying Hong [1*]

[1] Water Desalination and Reuse Center, Environmental Science and Engineering
[2] Red Sea Research Center, King Abdullah University of Science and Technology (KAUST), 4700 King Abdullah Boulevard, Thuwal 23955-6900, Saudi Arabia

* Corresponding author:
Pei-Ying Hong
Email: peiying.hong@kaust.edu.sa
Phone: +966-12-8082218

Table S1. GPS coordinates of sampling sites.

Name	Description	Longitude	Latitude
N1	North sites	39.0922°	21.6085°
N2		39.0923°	21.5995°
N3		39.0924°	21.5906°
N4		39.0932°	21.5816°
N5		39.0951°	21.5727°
N6		39.0684°	21.5906°
N7		39.0442°	21.5906°
N8		39.0201°	21.5905°
N9		38.9959°	21.5905°
S1	South sites	39.0833°	21.3413°
S2		39.0838°	21.3323°
S3		39.0863°	21.3235°
S4		39.0882°	21.3149°
S5		39.0883°	21.3060°
S6		39.0592°	21.3413°
S7		39.0207°	21.3413°
S8		38.9965°	21.3410°
NS1	North-South sites	39.0919°	21.4970°
NS2		39.0870°	21.4209°
T1	Transect	38.9436°	22.2798°
T2		38.9309°	22.1420°
T3		38.8486°	22.0287°
T4		38.9350°	21.9319°
T5		39.0014°	21.8141°
T6		39.0718°	21.6985°
T7		39.1489°	21.1000°
T8		39.0853°	21.6430°
KW1	KAUST Beach	39.0887°	22.2925°
KW2		39.0898°	22.2924°
KW3		39.0903°	22.2918°
KW4		39.0903°	22.2909°
TW1	Thuwal Beach	39.0877°	22.2765°
TW2		39.0876°	22.2751°
TW3		39.0872°	22.2743°
TW4		39.0868°	22.2732°

Table S2. Oligonucleotide primer sequences used for microbial source tracking and for quantification of pathogens.

Primer names	Target	Sequence (5'-3')	Amplicon size (bp)	Amplification factor
927F	Human-associated *Bacteroides vulgatus*	GGG CCC GCA CAA GCG G	110	1.61
Bvg1016R		ATG CCT TGC GGC TTA CGG C		
927F	Human-associated *Bacteroides fragilis*	GGG CCC GCA CAA GCG G	118	1.74
Bfrg1024R		TCA CAG CGG TGA TTG CTC A		
927F	Human-associated *Bacteroides uniformis*	GGG CCC GCA CAA GCG G	110	1.65
Bufm1018R		CTG CCT TGC GGC TGA CA		
C367F	Cow-specific *Bacteroidales*	GGA AGA CTG AAC CAG CCA AGT A	116	2.07
C467R		GCT TAT TCA TAC GGT ACA TAC AAG		
Pae-F	regA gene, *Pseudomonas aeruginosa*	CCGACTGACGCCAACGA	211	2.09
Pae-R		CGACCCTACCTCCCACGAAT		
Cc-F1	glyA gene, *Campylobacter coli*	CATATTGTAAAACCAAAGCTTATCGG	133	1.96
Cc-R1		AGTCCAGCAATGTGTGCAATG		
Abutz-F	rpoB gene, *Arcobacter butzleri*	ATA CTT TCT TGG TCT TGT GGT GTA	132	2.35
Agen-R		CCA CAA AGA CAC TCA TAA TCT TTT AC		

Table S3. Resistance patterns for enterococci and staphylococci isolates retrieved from the beach sands.

No. of antibiotics	No. of resistant isolates	Resistance Pattern
1	2 *E. faecium,*	K
	1 *E. casseliflavus*	Cz
	1 *E. faecalis*	C
	1 *S. epidermis*	A
2	1 *E. faecalis*	K, Cf
	2 *E. faecium*	K, Cz
3	1 *E. faecalis*	K, E, Cz
4	1 *E. faecium*	K, E, C, Mp
	1 *E. faecium*	K, Cz, C, Mp
5	1 *E. faecium*	K, E, Cz, C, Cf
	1 *E. faecium*	K, E, Cz, C, Mp

Abbreviations:

Ampicillin (A), chloramphenicol (C), ciprofloxacin (Cf), kanamycin (K), tetracycline (T), Erythromycin (E), Ceftazidime (Cz), Meropenem (Mp)

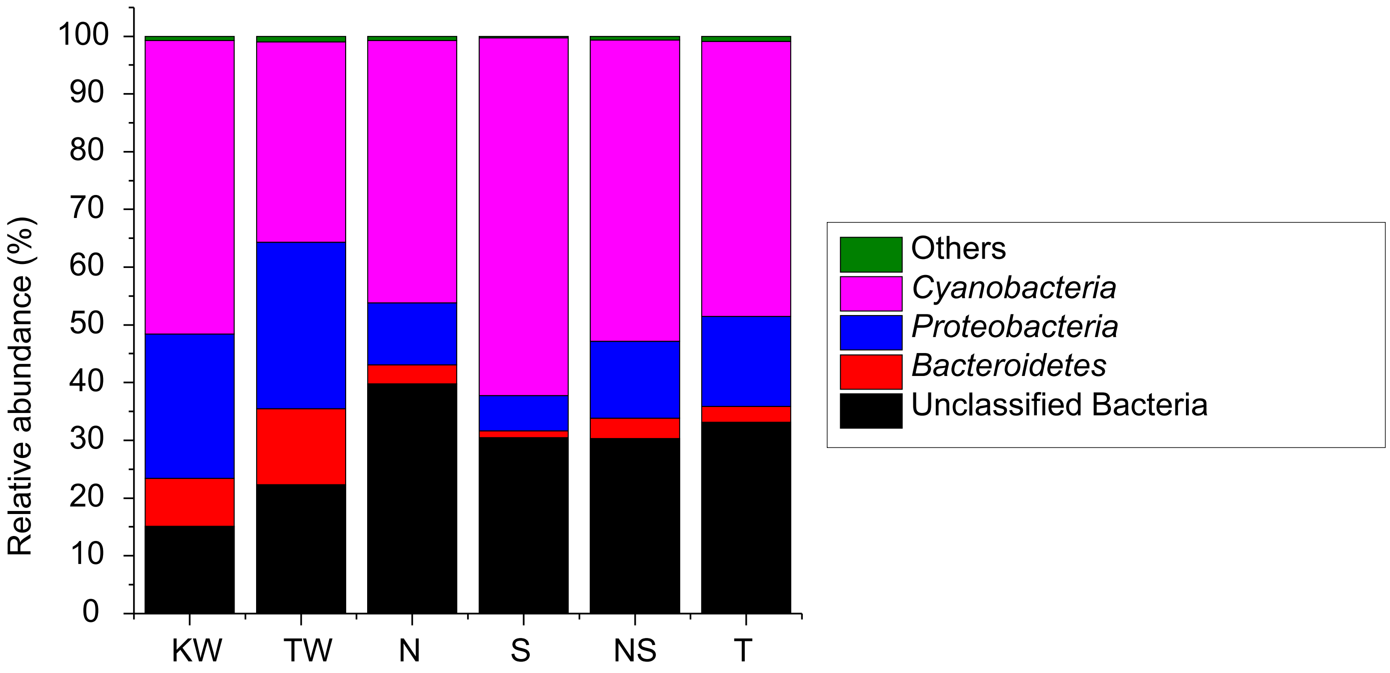

Figure S1. Predominant microbial phyla detected in the water samples.

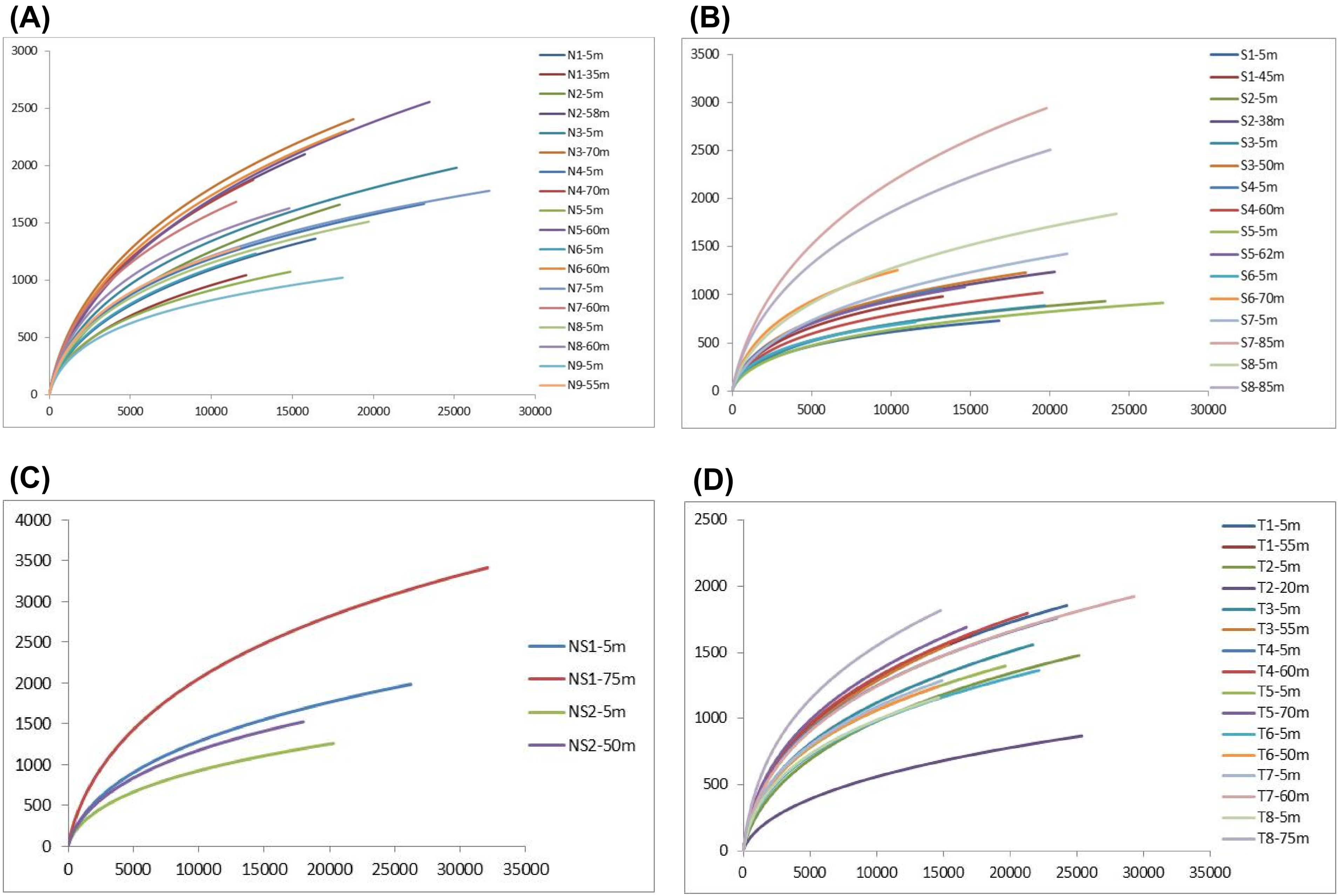

Figure S2. Rarefaction curves for all samples collected from **(A)** N sites, **(B)** S sites, **(C)** NS sites, and **(D)** T transects.

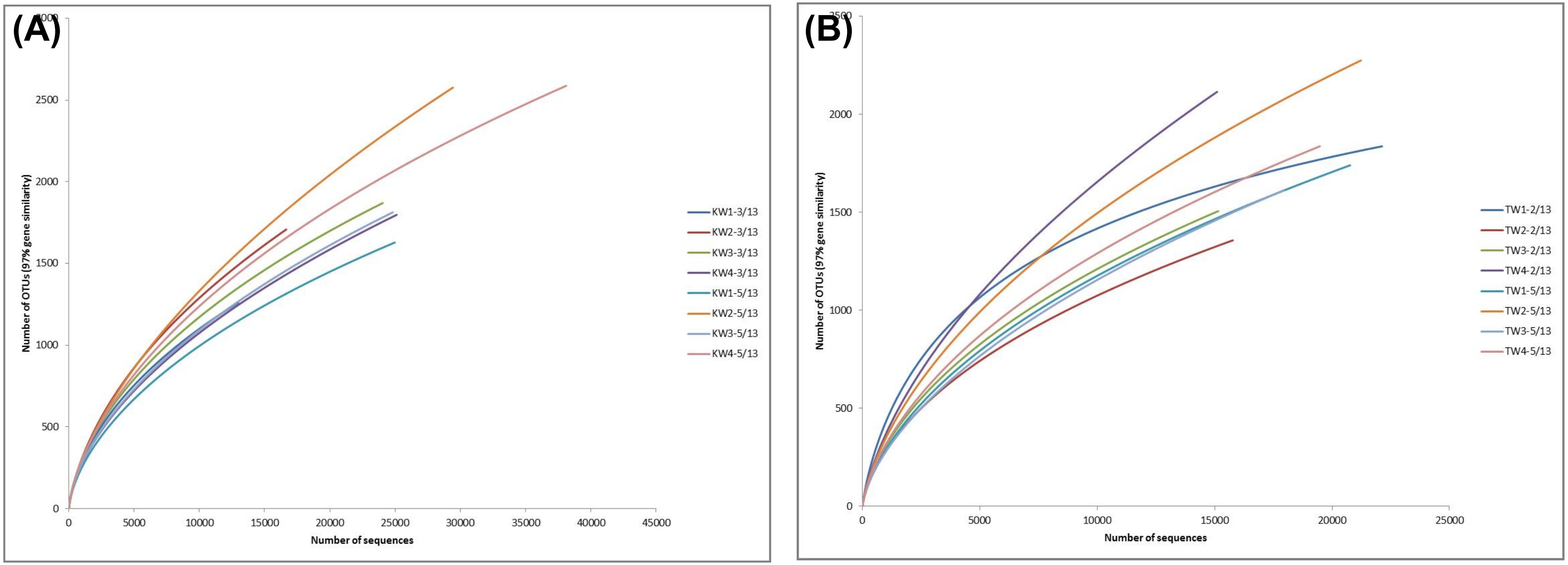

Figure S3. Rarefaction curves for all water samples collected from **(A)** KAUST beach, and **(B)** Thuwal beach
